# 858. Impact of Using an Order Set on PrEP Prescribing and Laboratory Monitoring in Primary Care

**DOI:** 10.1093/ofid/ofab466.1053

**Published:** 2021-12-04

**Authors:** Linda T Dao, Kathryn Medders, Lucas Hill

**Affiliations:** University of California San Diego Health, San Diego, CA

## Abstract

**Background:**

CDC 2017 pre-exposure prophylaxis (PrEP) guideline recommends laboratory monitoring at baseline and follow-up and specifies that a PrEP prescription should be written for once daily dosing with a supply of 90 days or less to ensure patients repeat HIV testing every 3 months. This presents an opportunity to utilize order sets in the electronic health record to improve PrEP prescribing habits and prescriber adherence to laboratory monitoring recommendations. This study assessed the impact of using an order set on the accuracy of PrEP prescriptions and the appropriateness of laboratory monitoring in the primary care setting.

**Methods:**

This was a retrospective, single-center, observational cohort study conducted at primary care clinics at a large academic health system. A total of 228 PrEP prescriptions from adults at least 18 years of age and that were written between April 1, 2018 through May 31, 2020 were assigned to the two comparator groups: 176 prescriptions ordered without an order set and 52 prescriptions ordered with an order set. The primary outcome was a composite of correct prescription details, defined as once daily dosing of PrEP for a 90-day supply or less. Secondary outcomes included the frequency of having an HIV antigen/antibody (Ag/Ab) test ordered within 3 months of the PrEP prescription, and the composite of appropriate baseline labs ordered for those newly starting PrEP.

**Results:**

Baseline characteristics are shown in Table 1. The primary outcome of correct prescription details occurred in 100% of PrEP prescriptions ordered with an order set compared to 65.9% of those ordered without an order set (P< 0.001). At least 1 HIV Ag/Ab test was appropriately repeated within 3 months for 65.4% of PrEP prescriptions ordered with an order set and 42.6% ordered without an order set (P=0.004). In those initiating PrEP, a composite of correct baseline labs ordered occurred with 14 (73.7%) new start prescriptions ordered with an order set versus 47 (42.7%) ordered without an order set (P=0.023).

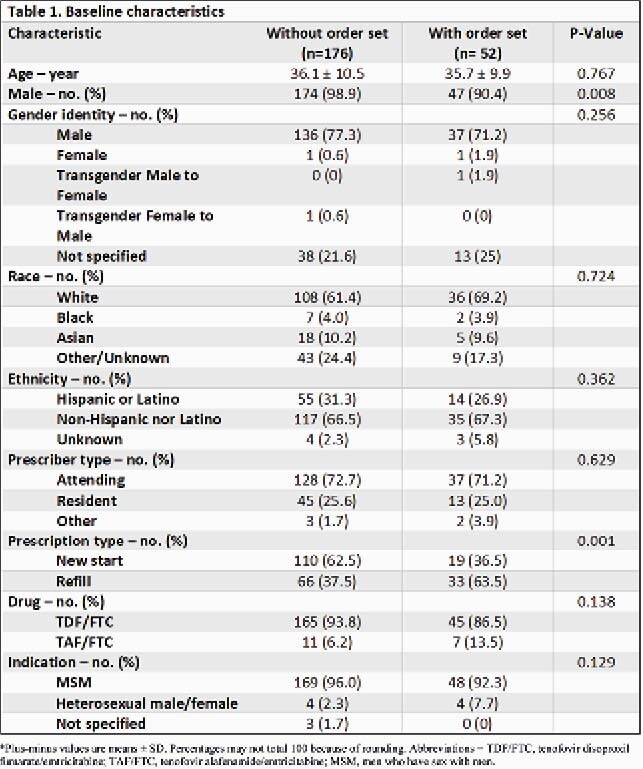

**Conclusion:**

When ordering PrEP, order set use significantly improved the accuracy of PrEP prescriptions and appropriateness of laboratory monitoring at baseline and at follow-up compared to no order set use at primary care clinics of a large academic health system.

**Disclosures:**

**All Authors**: No reported disclosures

